# The impact of antinuclear antibodies and complement levels in the prognosis of pregnant women within the antiphospholipid syndrome spectrum

**DOI:** 10.3389/fimmu.2026.1818605

**Published:** 2026-06-05

**Authors:** Sara Garcia Bravo, Víctor M. Martínez-Taboada, Sara del Barrio-Longarela, Ana Merino, Alejandra Comins-Boo, Marcos López-Hoyos, José L. Hernández

**Affiliations:** 1 Division of Rheumatology, Hospital Marqués de Valdecilla, Santander, Spain; 2 Departamento de Medicina y Psiquiatría, Universidad de Cantabria, Santander, Spain; 3 Immunopathology Group, Instituto de Investigación Marqués de Valdecilla (IDIVAL), Santander, Spain; 4 Division of Obstetrics and Gynecology, Hospital Marqués de Valdecilla, Santander, Spain; 5 Immunology Department, Hospital Universitario Marqués de Valdecilla, Santander, Spain; 6 Departamento de Biología Molecular. Universidad de Cantabria, Santander, Spain; 7 Department of Internal Medicine, Hospital Marqués de Valdecilla, Santander, Spain

**Keywords:** antinuclear antibodies, antiphospholipid antibodies, antiphospholipid syndrome, autoantibodies, complement, fetal loss, obstetric morbidity, preeclampsia

## Abstract

**Introduction:**

To assess the prevalence and clinical impact of antinuclear antibodies (ANA) and low complement levels on fetal–maternal outcomes in pregnant women within the spectrum of obstetric antiphospholipid syndrome (APS).

**Methods:**

A total of 285 ever-pregnant women with APS-related obstetric morbidity were included. Among them, 85 fulfilled APS classification criteria, 139 were classified as non-criteria APS (NC-APS), and 61 as seronegative APS (SN-APS). Patients with other autoimmune diseases were excluded. Adverse pregnancy outcomes (APO) included early pregnancy loss, fetal death, preeclampsia, abruptio placentae, and preterm birth. Successful pregnancy was defined as the achievement of a live birth. ANA positivity and complement levels were analyzed in relation to obstetric outcomes and treatment prescription patterns.

**Results:**

Sixty patients (22.3%) were ANA positive. ANA positivity was associated with a lower number of pregnancies and live births, with the lowest ANA titers observed in the SN-APS group. Fifteen patients (6.3%) presented low complement levels. Hypocomplementemia was associated with a higher incidence of preeclampsia and early miscarriages, particularly among NC-APS patients. Additionally, all serological abnormalities were significantly associated with increased prescription of antimalarial therapy during pregnancy.

**Discussion:**

ANA positivity and hypocomplementemia were associated with poorer obstetric outcomes in women within the spectrum of obstetric APS. These serological abnormalities may identify a subgroup of patients with a higher-risk obstetric profile and greater need for intensified therapeutic management, including the use of antimalarial treatment during pregnancy.

## 1 Introduction

Antiphospholipid syndrome (APS) is an autoimmune disease characterized by thrombotic and/or obstetric events, associated with the presence of antiphospholipid antibodies (aPL) (1). Diagnosing APS requires both clinical and serological criteria, but patients who do not strictly meet the classification criteria may present with what have been called “clinical manifestations related to APS”, or with an inconclusive serological profile not included within the criteria definition. This is especially relevant in the subgroup of patients with obstetric APS (2).

Although there is a clear association between obstetric complications and the presence of antiphospholipid antibodies (aPL), women of reproductive age may present other associated comorbidities that may complicate their pregnancy wishes (3). More specifically, in addition to cardiovascular risk factors, such as obesity, smoking, or hypertension, other diseases, such as thyroid diseases, local uterine disorders, or inherited thrombophilia (IT), have been related to the possibility of developing adverse events during pregnancy (4–7).

In addition to the aPL, a growing number of studies have explored the roles of antinuclear antibodies (ANA) and especially the complement system in pathophysiology and clinical impact on obstetric APS. ANA has emerged as a potential risk modulator in patients with APS and specifically, obstetric APS. Ottavi et al. (8) reported that ANA positivity was associated with a higher risk of thrombotic relapses, highlighting the need to include ANA status in comprehensive APS risk assessment. Furthermore, Ricard et al. (9) showed that ANA+ in primary APS was linked to greater obstetric morbidity and thrombotic recurrence. Noteworthy, in a recent large multicenter study including 430 aPL+ patients, ANA-negative women had better pregnancy outcomes (10). Complement activation is also a critical factor in pregnancy outcomes in women with APS (11). Thus, multicenter studies indicated that low C3 and C4 levels in the first trimester predicted premature birth, low birth weight, and other severe obstetric complications (12). Furthermore, both human and animal studies have shown that complement inhibition can be protective against aPL-induced thrombosis and fetal loss (11, 13, 14).

Most available studies have focused either on patients with systemic autoimmune diseases, especially systemic lupus erythematosus, or on highly selected cohorts fulfilling strict APS classification criteria (8–14). Consequently, limited information is available regarding the prevalence and clinical significance of ANA positivity and hypocomplementemia across the whole clinical spectrum of obstetric APS, including non-criteria APS and seronegative APS. Furthermore, the potential implications of these serological abnormalities for therapeutic decision-making remain insufficiently explored. Identifying additional immunological profiles associated with poorer obstetric outcomes could improve risk stratification and support a more individualized management approach, particularly regarding the use of immunomodulatory therapies such as hydroxychloroquine.

Taking into account these considerations and the paucity of data published to date, our study aimed to assess the prevalence and impact of ANA positivity and low complement levels on fetal-maternal outcomes in pregnant women within the spectrum of obstetric APS.

## 2 Materials and methods

### 2.1 Study participants

**Figure 1:**
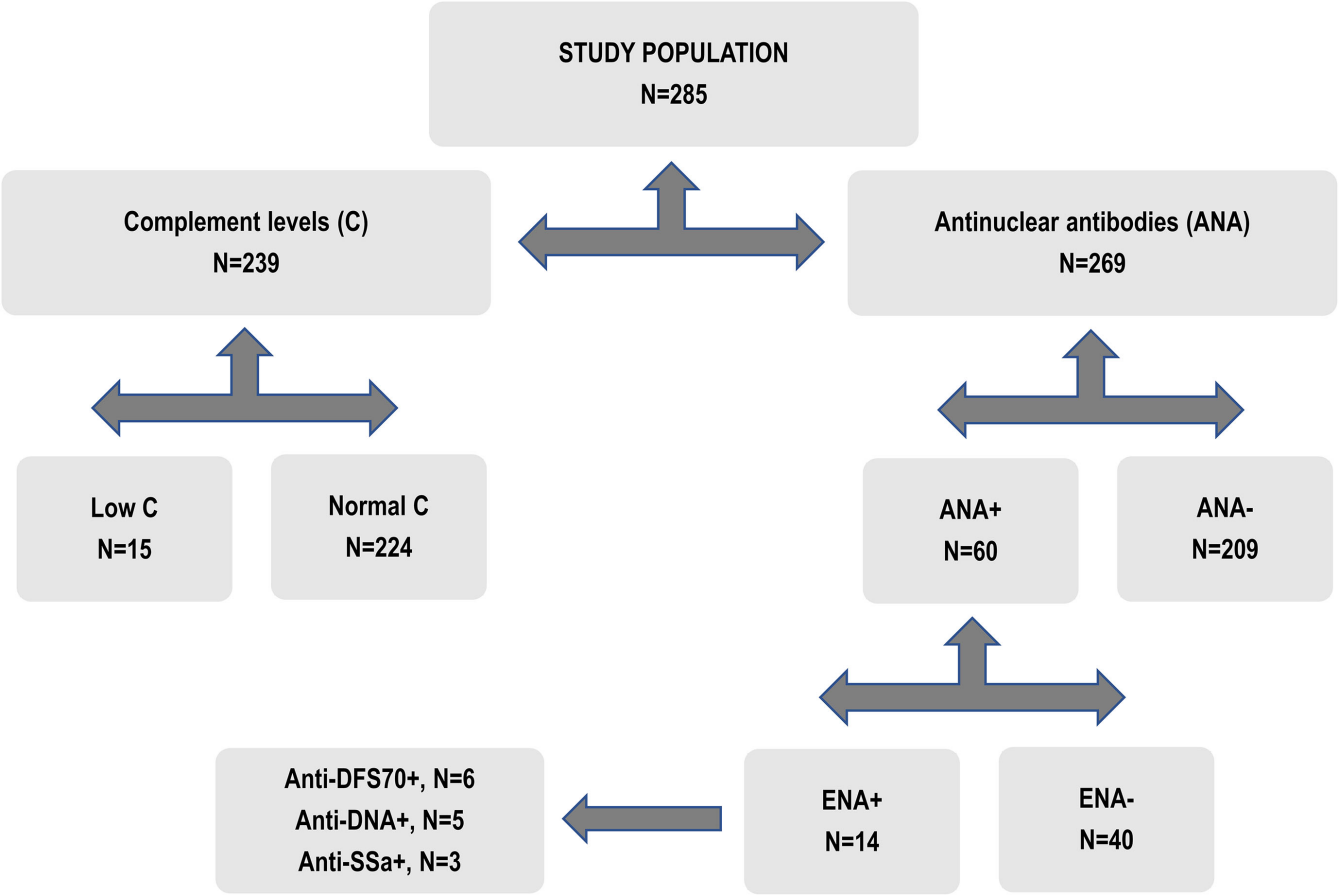
Study group algorithm.

This retrospective cohort study included 285 women followed at the Autoimmune Diseases Pregnancy Clinic, a multidisciplinary unit of a teaching tertiary care hospital, between 2005 and 2023. Inclusion criteria were: a) patients included in previously well-defined clinical-serological subgroups (15), b) with ANA and/or complement study available, and c) with at least one clinical pregnancy (**Figure 1**). Two hundred and twenty-three patients had ANA and complement levels performed simultaneously, while 46 patients only had ANA determination, and 16 had complement determination alone. As shown in **Supplementary Table 1**, patients were categorized into the following groups: a) Criteria APS (n=85): patients were classified according to the Sidney classification criteria (1); b) non-criteria APS-(NC) (n=139): patients who do not meet strict clinical and serological classification criteria for the disease. According to Alijotas-Reigh et al. (2), these patients were divided into the following subgroups: Subgroup A (n=27): non-criteria obstetric morbidity related to APS and inconclusive serology; Subgroup B (n=50): clinical manifestations included in the criteria and inconclusive serology; and Subgroup C (n=62): non-criteria obstetric morbidity related to APS and serology included in the classification criteria.; c) seronegative APS-(SN) (n=61): clinical manifestations included in the criteria and persistently negative serology. Women who fulfilled the classification criteria for rheumatic autoimmune diseases other than APS were excluded.

The information collected from individual cases was completely anonymized, and the study was approved by the Ethics Committee of Cantabria (internal code: 2025.004).

### 2.2 Data collection

Data were collected using a prespecified standardized questionnaire in a computerized database. We assessed the following clinical variables (16):

- Demographic and general characteristics: age, sex, body mass index (BMI), current/past tobacco use, high blood pressure (equal or greater than 140/90 mm Hg or being on antihypertensive agents), dyslipidemia (serum total cholesterol or triglyceride levels greater than 230 mg/dl and 150 mg/dl respectively or being on lipid-lowering drugs), diabetes mellitus (17), past or present family (<50 years) or personal history of thrombotic disease.

- Comorbidities: three main entities associated with pregnancy outcomes were also recorded: a) inherited thrombophilia (7); b) thyroid disease (history of hypo/hyperthyroidism or the presence of confirmed specific autoantibodies) (5); c) obstetric comorbidity (local uterine abnormalities, endometriosis, and polycystic ovary syndrome) (6).

### 2.3 Autoantibody assessment

#### 2.3.1 aPL

The presence of the following antibodies and aPL isotypes was quantified by commercial enzyme immunoassay in solid phase (ELISA; Orgentec Diagnostika GmbH, Mainz, Germany): anticardiolipin antibodies (aCL) and anti-beta2 glycoprotein I antibodies (AB2GPI) of the IgG and IgM isotypes. The results are reported as quantitative and semiquantitative values. Thus, aCL are quantified in GPL (aCL IgG) or MPL (aCL IgM) according to the standard curve constructed in each test with 5 dilution points of the Harris/Sapporo standards. AB2GPI are quantified as U/ml. Only medium-high titers of aPL were considered positive. The criteria recommended by the International Society of Thrombosis and Hemostasis (ISTH) Scientific and Standardization Committee (ISTH) for the standardization of lupus anticoagulant/antiphospholipid antibodies (LA/APA) were applied for the characterization of LA (18–20). Inconclusive serology was defined as persistent low titer aCL or AB2GPI and/or intermittent AL, aCL, or AB2GPI.

#### 2.3.2 Antinuclear antibodies

IIF assay on HEp-2 cells

Serum was diluted 1:160 with phosphate-buffered saline (PBS), which was considered the screening dilution. A Zeiss fluorescence microscope with incident mercury light illumination and filters for activation/emission of fluorescein isothiocyanate (FITC) was used. Slides with fixed HEp-2 cells served as a source of nuclear antigens (Biosystems, Barcelona, Spain). FITC-conjugated rabbit anti-human IgG was used as the secondary antibody (Biosystems, Barcelona, Spain). Incubations, washing steps, and mounting microscope slides were done following the manufacturer´s instructions. The slides were inspected under the fluorescence microscope at 400 magnification. Nuclear, cytoplasmic, and mitotic HEp-2 patterns were considered, and the nomenclature for ANA detected using the IIF assay on HEp-2 cells was performed according to the International Consensus on ANA Patterns (ICAP) (21, 22).

Extractable nuclear antigens (ENAs) were measured using an automated multiparticle system (PMAT) by an automated immunofluorescence test (Aptiva, Werfen, San Diego, USA). The system quantitatively determines anti-dsDNA antibodies in U/ml (cut-off < 40 U/ml) and qualitatively (negative/positive) for Scl-70, centromere B, Ro (SSA-52, SSA-60), RNP, Sm, Jo1, SSB/La, antiDFS70, and ribosomal protein P, following the manufacturer’s instructions.

#### 2.3.3 Complement levels

C3 and C4 levels were quantified in serum by immunoturbidimetry using Optilite equipment (Binding Site, Birmingham, UK) and expressed in mg/dl. Reference values: for C3 were 40–120 mg/dl, and for C4, 8–20 mg/dl.

### 2.4 Pregnancy morbidity definitions

* Obstetric manifestations: a) Sidney criteria (1); b) Non-criteria obstetric morbidity related to APS: 1–2 early pregnancy losses (<10 weeks), preterm birth (between 34 and 36 + 6 weeks), late preeclampsia (PE) (>34 weeks), abruptio placentae, and unexplained *in vitro* fertilization failures (IVF) (>2) (23).

* Pregnancy loss: early pregnancy loss (<10 weeks) and/or fetal death (>10 weeks).

* Adverse pregnancy outcome (APO): early pregnancy loss, fetal death, preeclampsia, abruptio placentae, and preterm birth (<37 weeks).

* A successful pregnancy was defined as the achievement of a live newborn.

### 2.5 Statistical analysis

Results were expressed as numbers (percentage), mean ± standard deviation (SD), or median and interquartile range (IQR), as appropriate. Student’s t-test, Mann-Whitney U-test, or one-way ANOVA were used to compare quantitative variables, and Chi-squared or Fisher test, to compare categorical data. A two-tailed p-value <0.05 was considered statistically significant in all the calculations. IBM SPSS 28.0 was used for statistical analyses (Armonk, NY: IBM Corp).

## 3 Results

### 3.1 General features of the study cohort

During the study period, 285 consecutive patients fulfilled the inclusion criteria (**Figure 1** and **Supplementary Table 1**). The main characteristics of the study cohort are shown in **Table 1**.

**Table 1:** Demographic characteristics, cardiovascular risk factors, and main comorbidities in the different study groups.

	Total N= 285	Criteria APS N= 85	Non Criteria APS N= 139	Seronegative APS N= 61
Age, *yrs ± SD*	34 ± 5.5	33.9 ± 5.7	33.9 ± 5.7	34.3 ± 4.9
Time to diagnosis (days), *median [IQR]*	23 [12-53]	29 [12–57]	21 [11–44]*	34 [16–67]*
Follow-up, *months*, *median [IQR]*	36 [11-94]	64 [21-161]^#§^	31 [11-69]^#^	21 [4-60]^§^
CV risk factors, *N(%)*	147 (51.6)	54 (63.5)^¶^	64 (46.5)	29 (47.5)
- Obesity	47 (18.4)	22 (29.3)^£^	19 (15.3)	6 (10.5)
- Smoking	96 (33.7)	35 (41.2)^¥^	44 (31.7)	17 (27.9)
- High blood pressure	23 (8.1)	7 (8.2)	11 (7.9)	5 (8.2)
- Diabetes	8 (2.8)	2 (2.4)	3 (2.2)	3 (4.9)
- Dyslipidemia	17 (6.0)	7 (8.2)**	10 (7.2)	0
Comorbidities, *N (%)*				
- Inherited thrombophilia	32 (11.2)	10 (11.8)	11 (7.9)	11 (18)
- Thyroid disease	57 (20)	16 (18.8)	28 (20.1)	13 (21.3)
- Obstetric comorbidity	41 (14.4)	8 (9.4)	24 (17.3)	9 (14.8)

1 APS, antiphospholipid syndrome; *APS-NC vs APS-SN, p<0,05; ^#^Criteria APS vs APS-NC, p<0,0001; ^§^Criteria APS vs APS-SN, p<0,0001; ^¶^p value for trend: 0.04; ^£^p value for trend: 0.004; ^¥^p value for trend: 0.08; **p value for trend: 0.05.

The mean age of the overall group was 34 ± 5.5 years, and the patients were followed up for 36 [11-94] months. Overall, and as previously described (16) he prevalence of cardiovascular risk factors ranged from 46% to 64% and was especially frequent in patients with APS. In addition, the most frequent comorbidities with a potential impact on the obstetric outcome, such as IT, thyroid disease, or obstetric comorbidities, were also frequent in all study groups. After diagnosis, most of them received standard of care (SoC) treatment with low-dose aspirin (LDA) and/or low-molecular-weight heparin (LMWH) during subsequent pregnancies (**Supplementary Tables 2**, **3**) (24–27).

### 3.2 Prevalence of ANA and low complement levels

In 60 of the 269 (22.3%) patients who underwent ANA testing, the results were positive by indirect immunofluorescence (IIF) (**Table 2**). Although no statistically significant differences were observed across the study groups, patients with seronegative APS (APS-SN) had a lower frequency of ANA+ (p=0.09). Overall, patients had moderate or low titers. It should be noted that the majority of patients with APS-SN (85.7%) had titers ≤1/160 (p=0.13).

**Table 2:** Detailed serologic features in the different study groups.

	Total	Criteria APS	Non-Criteria APS	Seronegative APS
aPL profile, *N (%)*				
- Single+	98 (34,4)	55 (64.7)	43 (30.9)	–
- Doble+	37 (13)	19 (22.4)	18 (12.9)	–
- Triple+	16 (5.6)	11 (12.9)	5 (3.6)	–
ANA + titer, *N (%)*	60 (22.3)	21 (25.9)*	32 (23.9)	7 (13)*
- ≤ 1/160	30 (50)	9 (42.9)^§^	15 (46.9)	6 (85.7)^§^
- 1/320	12 (20)	6 (28.6)	6 (18.8)	0
- 1/640	6 (10)	2 (9.5)	3 (9.4)	1 (14.3)
- ≥ 1/1280	12 (20)	4 (19)	8 (25)	0
Low C3 and/or C4, *N (%)*	15 (6.3)	8 (10.5)^¥^	5 (4.1)^¥^	2 (4.8)

APS, antiphospholipid syndrome; aPL, antiphospholipid antibodies; ANA, antinuclear antibodies; *Criteria APS vs APS-SN, p=0.07 ^§^Criteria APS vs APS-SN, p=0.13; ^¥^Criteria APS vs APS-NC, p=0.07.

ENAs were determined in 54 of the 60 ANA+ patients (90%) and were only positive in 14. Anti-DFS70 was the most frequent antibody found (N = 6), followed by anti-DNA (N = 5) and anti-SSa (N = 3). None of these patients presented symptoms suggestive of another systemic autoimmune disease at diagnosis or during follow-up (**Figure 1**).

Fifteen of the 239 (6.3%) patients who underwent complement testing had decreased levels of C3 and/or C4 (**Table 2**).

**Figure 2:**
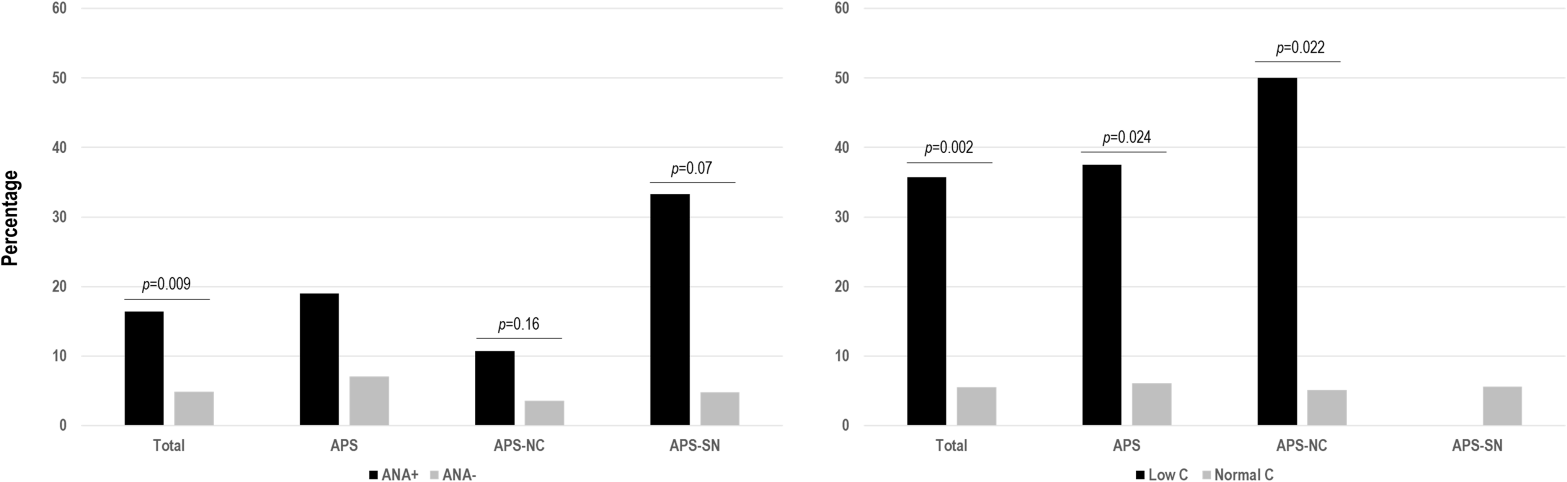
Antimalarial treatment in the different study groups according to serological profile: C, complement levels; ANA, antinuclear antibodies; APS, antiphospholipid syndrome; NC, non-criteria; SN, seronegative.

Although no statistically significant differences were observed between the study groups, patients with APS criteria presented a higher frequency of hypocomplementemia (p=0.07). As shown in **Figure 2**, ANA and complement levels were determined in 223 patients. It is noteworthy that six ANA+ patients had low complement levels, whereas the remaining nine patients with hypocomplementemia tested negative for ANA. None of the patients with ANA+ and hypocomplementemia had positive anti-DNA results or any other clinical signs suggestive of systemic lupus erythematosus (SLE). Furthermore, the only patient with decreased C3 and C4 levels had anti-DFS70 antibodies, and one patient with decreased C3 levels had also anti-SSa antibodies.

### 3.3 Impact of ANA on the number of pregnancies and adverse pregnancy outcomes

As shown in **Table 3**, overall, ANA+ patients had a lower number of pregnancies (p=0.049), especially those in the criteria APS group (p=0.19). ANA+ patients also had a lower live birth rate, mainly those in the APS-SN group (p=0.17). Notably, within the overall cohort, the rate of live births without treatment was significantly higher among patients with lower ANA titers (71.4% vs. 28.6%; p=0.02). No additional significant differences in obstetric outcomes were identified, either in the overall population or across the three study groups (primary APS, non-criteria APS, and seronegative APS).

**Table 3:** Number of pregnancies, number of live births, and adverse pregnancy outcomes (APO) in patients with and without antinuclear antibodies (ANA).

	Total N=269		Criteria APS N=81		Non-Criteria APS N=134		Seronegative APS N=54	
	ANA + N=60	ANA - N=209	ANA + N=21	ANA - N=60	ANA+ N=32	ANA - N=102	ANA+ N=7	ANA- N=47
Number of pregnancies, *median [IQR]*	3 [2-5]*	4 [3-5]*	4 [2.5-4.5]^#^	4 [3-5] ^#^	3 [2-5]	4 [2-5]	4 [4-6]	5 [4-6]
Number of live births, *median [IQR]*	1 [1-2]	2 [1-2]	1 [1-2]	2 [1-2]	2 [1-2]	2 [1-2]	1 [1-1]^§^	2 [1-2]^§^
APO total, *median [IQR]*	2 [1-3]^¶^	3 [2-3]^¶^	3 [2-3]	3 [2-4]	2 [1-3]	2 [1-3]	3 [3-4]	3 [3-4]
APO total, *N (%)*	53 (88.3)**	201 (96.2)**	16 (76.2)^##^	55 (91.7) ^##^	30 (93.8)	99 (97.1)	7 (100)	47 (100)
Abortion <10 weeks	16 (26.7)	41 (19.6)	14 (66.7)	40 (66.7)	24 (75)	82 (80.4)	6 (85.7)	46 (97.9)
Fetal death >10 weeks	10 (16.7)	37 (17.7)	5 (23.8)	23 (38.3)	3 (9.4)	8 (7.8)	2 (28.6)	6 (12.8)
Preterm <37 weeks	9 (15)	34 (16.3)	4 (19)	16 (26.7)	5 (15.6)	13 (12.7)	0	5 (10.6)
Abruptio placentae	1 (1.7)	9 (4.3)	1 (4.8)	1 (1.7)	0^¥^	7 (6.9%)^¥^	0	1 (2.1)
Preeclampsia	8 (13.3)	25 (12)	2 (9.5)	4 (6.7)	6 (18.8)	18 (17.6)	0	3 (6.4)

APS, antiphospholipid syndrome; ANA, antinuclear antibodies; APO, adverse pregnancy outcome.

*Total: p=0.049; ^#^Criteria APS: p=0.19; ^§^APS-SN: p=0.17; ^¶^Total: p=0.09; **Total: p=0.048; ^##^Criteria APS: p=0.12; ^¥^APS-NC: p=0.19.

Interestingly, ANA+ patients had a lower percentage of APO (p=0.048), as well as a trend toward a lower number of total APO (p=0.09). Furthermore, a trend toward a lower incidence of placental abruption was observed in ANA+ patients in the non-criteria APS (APS-NC) group (p=0.19).

### 3.4 Impact of hypocomplementemia on the number of pregnancies and adverse pregnancy outcomes

As shown in **Table 4**, overall, patients with hypocomplementemia showed a higher frequency of preeclampsia (PE) compared to patients with normal C levels (p=0.12). However, the impact of complement levels was more significant in the group of patients with APS-NC. In this regard, patients with hypocomplementemia showed a tendency toward a lower number of pregnancies (p=0.17), as well as a higher frequency of early miscarriages (p=0.01) and PE (p=0.047).

**Table 4:** Number of pregnancies, number of live births, and adverse pregnancy outcomes (APO) in patients with and without low complement levels.

	Total N=239		Criteria APS N=76		Non Criteria APS N=121		Seronegative APS N=42	
	Low C N=15	Normal C N=224	Low C N=8	Normal C N=68	Low C N=5	Normal C N=116	Low C N=2	Normal C N=40
N° of pregnancies, *median [IQR]*	4 [3-5]	4 [3-5]	4 [3.25-5]	4 [3-5]	3 [1-3.5]*	3 [2-5]*	4.5 [2-6]	5 [4-6]
Number of live births, *median [IQR]*	2 [1-2]	2 [1-2]	2 [1-2]	1.5 [1-2]	1 [1-3.5]	2 [1-2]	1.5 [1-2]	2 [1-2]
APO total, *median [IQR]*	3 [1-3]	2 [2-3]	3 [2.25-3.75]	3 [2-4]	1 [0.5-2]	2 [1-3]	3 [3-3]	3 [3-4]
APO total, *N (%)*	13 (86.7)	211 (94.2)	7 (87.5)	60 (88.2)	4 (80)	111 (95.7)	2 (100%)	40 (100%)
Abortion <10 weeks	10 (66.7)	172 (76.8)	7 (87.5)	43 (63.2)	4 (80)^#^	24 (20.7)^#^	2 (100)	37 (92.5)
Fetal death >10 weeks	3 (20)	46 (20.5)	3 (37.5)	25 (36.8)	0	10 (8.6)	0	11 (27.5)
Preterm <37 weeks	3 (20)	43 (19.2)	2 (25)	19 (27.9)	1 (20)	18 (15.5)	0	6 (15)
Abruptio placentae	0	10 (4.5)	0	2 (2.9)	0	7 (6)	0	1 (2.5)
Preeclampsia	4 (26.7)^£^	28 (12.5)^£^	1 (12.5)	4 (5.9)	3 (60)^§^	20 (17.2)^§^	0	4 (10)

APS, antiphospholipid syndrome; C, complement; APO, adverse pregnancy outcome;.

*APS-NC: p=0.17; ^#^APS-NC: p=0.01; ^£^Total: p=0.12; ^§^APS-NC: p=0.047.

We also have analyzed a more stringent APO, such as preterm birth before 34 weeks, including 18 patients (10 primary APS, 5 NC-APS, and 3 seronegative APS), and we did not find any significant difference in the presence of either ANA or hypocomplementemia.

### 3.5 Do patients with ANA+ and/or hypocomplementemia receive different therapy?

In the different study groups and according to the presence or absence of ANA+, and hypocomplementemia, the main treatments used during pregnancy were analyzed (**Tables 5**, **6**).

**Table 5:** Number of pregnancies, number of live births and adverse pregnancy outcomes (APO) in patients with and without antinuclear antibodies (ANA).

	Total N= 269		Criteria APS N=81		Non criteria APS N=134		Seronegative APS N=54	
	ANA + N=60	ANA - N=209	ANA + N=21	ANA - N=60	ANA + N=32	ANA - N=102	ANA + N=7	ANA - N=47
Standard treatment, *N (%)*								
**- LDA monotherapy**	19 (31.7)	16 (26.7)	4 (19)	16 (26.7)	12 (37.5)	40 (40)	3 (42.9)	17 (37)
**- LDA+LWMH**	33 (55)	108 (51.9)	14 (66.7)	40 (66.7)	15 (46.9)	47 (46.5)	4 (57.1)	21 (44.7)
Additional treatments, *N (%)*								
**- Corticosteroids**	10 (18.9)	23 (12.5)	2 (10)	3 (5.3)	5 (18.5)*	6(7.1)*	3 (50)	14 (32.6)
**- Antimalarials**	9 (16.4)^£^	9 (4.9)^£^	4 (19)	4 (7.1)	3 (10.7)^#^	3 (3.6)^#^	2 (33.3)^§^	2 (4.8)^§^
**- IVIG**	0	6 (3.9)	0	1 (2.1)	0	2 (2.8)	0	3 (8.8)

APS, antiphospholipid syndrome; ANA, antinuclear antibodies; LDA, low dose aspirin; LWMH, low molecular weight heparin. IVGG, intravenous immunoglobulins. *Non-Criteria APS: *p* = 0.13; ^£^Total: *p* = 0.009; ^#^Non-Criteria APS: *p* = 0.16; ^§^Seronegative APS: *p* = 0.07.

**Table 6:** Number of pregnancies, number of live births, and adverse pregnancy outcomes (APO) in patients with and without low complement levels.

	Total N=239		Criteria APS N=76		Non criteria APS N=121		Seronegative APS N=42	
	Low C N=15	Normal C N=224	Low C N=8	Normal C N=68	Low C N=5	Normal C N=116	Low C N=2	Normal C N=40
Standard treatment, *N (%)*								
- LDA monotherapy	3 (20)	76 (34.5)	0	15 (22.1)	2 (40)	49 (43)	1 (50)	12 (31.6)
- LDA+LWMH	10 (66.7)	115 (51.6)	7 (87.5)	47 (69.1)	2 (40)	50 (43.5)	1 (50)	18 (45)
Additional treatments, *N (%)*								
- Corticosteroids	3 (21.4)	24 (11.9)	1 (12.5)	4 (6)	1 (25)	9 (9.2)	1 (80)	11 (29.9)
- Antimalarials	5 (35.7)*	11 (5.5)*	3 (37.5)^§^	4 (6.1)^§^	2 (50)^#^	5 (5.1)^#^	0	2 (5.6)
- IVIG	0	5 (3.1)	0	1 (1.9)	0	2 (2.5)	0	2 (6.9)

APS, antiphospholipid syndrome; C, complement; LDA, low dose aspirin; LWMH, low molecular weight heparin. IVIG, intravenous immunoglobulins. *Total: *p* = 0.002; ^§^Criteria APS: *p* = 0.024; ^#^Non-Criteria APS: *p* = 0.022.

Treatment was divided into standard treatment (LDA monotherapy or the combination of LDA and LMWH) and additional treatments that included low-dose corticosteroids, antimalarials (primarily hydroxychloroquine), and polyclonal human gamma globulin.

No significant differences were observed among the study groups concerning SoC or additional treatments across the different serological profiles, except for antimalarial use. As shown in **Figure 2**, patients with ANA positivity and/or hypocomplementemia were more frequently treated with antimalarial agents. This association was particularly pronounced in patients with APS and APS-NC who had hypocomplementemia.

## 4 Discussion

In the present study, we analyzed the impact of ANA positivity and serum complement levels on obstetric outcomes and the need for additional treatment in patients across the clinical spectrum of APS. Overall, approximately a quarter of patients were ANA+, while only 6% of them had hypocomplementemia. In line with previous studies, ANA positivity and hypocomplementemia were associated with the obstetric outcome. Furthermore, these associated serological abnormalities in patients across the clinical spectrum of APS were associated with a higher prescription of adjuvant antimalarial therapy.

The impact of ANA positivity on the development of obstetric complications and its possible effect on fertility has been previously studied in women without known autoimmune diseases, with conflicting results (28, 29). Whereas a meta-analysis of six studies reported an adverse association between ANA positivity and both IVF and the risk of recurrent pregnancy loss (RPL), data in terms of late pregnancy complications were inconclusive (29). In contrast, in a case-control study, no significant ANA differences were found between RPL cases and controls, suggesting ANA alone may not predict RPL (28). Furthermore, a recent multicenter study reported that ANA detection after obstetric complications helped to diagnose autoimmune diseases early, though it did not influence future pregnancy outcomes (30).

In patients with APS not associated with other autoimmune diseases, information is more limited. While some studies have not found an association between ANA positivity and the development of clinical manifestations such as thrombosis or obstetric complications (10). Others have found an increased risk of thrombotic complications in ANA+ patients (8, 9) and also an increase in obstetric morbidity (9). Furthermore, in a recent study by Cecchi et al. (10) ANA-negative women had better pregnancy outcomes. ANA-negative patients presented with a higher number of pregnancies and live births when compared with the ANA+ subgroup. These findings are consistent with our results and further support the notion that the presence of autoantibodies beyond aPL may have clinical relevance. Regarding the serological findings in the different study groups, it is not surprising that patients with APS-SN had a lower frequency and also lower titers of ANA. These data reinforce the seronegative nature of this subgroup, supporting the idea that they do not present a clear systemic autoimmunity, and their diagnosis is based primarily on clinical findings. ANA+ patients had a lower percentage of APOs, along with a trend toward a reduced total number of APOs, which appears to be attributable to a lower number of pregnancies in this group. Since the analysis was performed on patients and not on pregnancies, this data should be taken with caution. An alternative explanation, as suggested by Cecchi et al. (10), could be that ANA+ patients are more frequently treated with antimalarials, and these drugs might also decrease the number of obstetric complications, as has been previously described (31–33). An interesting finding was that the most frequently detected ENA was anti-DFS70. Among ANA-positive individuals, isolated anti-DFS70 antibodies may represent a clinically useful biomarker for distinguishing systemic autoimmune diseases from subjects without such conditions in those with positive ANA by indirect immunofluorescence (34). In our series, the vast majority of patients with anti-DFS70 antibodies belong to the NC-APS and SN-APS groups, suggesting that both groups have a lower autoantibody load.

The complement system has emerged as a pivotal player in the pathogenesis of obstetric APS, influencing both maternal and fetal outcomes. Experimental animal models have been instrumental in elucidating the mechanisms by which complement activation contributes to pregnancy complications. Thus, some studies have found that complement activation is essential for the development of aPL-induced placental insufficiency and fetal loss (35, 36). Importantly, complement inhibition, through agents like hydroxychloroquine, has shown protective effects in these models, thereby suggesting potential therapeutic implications for pregnancies affected by obstetric APS (36). Furthermore, *in vitro* models have introduced new treatment options that mitigate complement activation, thereby reducing the development of clinical manifestations, including fetal outcomes (37). A growing body of clinical research corroborates these findings. Observational cohort studies have consistently linked low serum levels of complement components C3 and C4 with adverse pregnancy outcomes in women who tested positive for aPL, particularly those with triple positivity. For instance, Esteve-Valverde et al. (12) reported that low complement levels were predictive of preterm birth and low birth weight, while Fredi et al. (38) found similar associations in early gestation. Complement activation biomarkers -such as elevated plasma Bb and C5b-9- have also been identified as early predictors of adverse events in patients with SLE and/or aPL+ (39).

Only 6.3% of patients who underwent complement testing had decreased levels of C3 and/or C4. It is striking that this figure is significantly lower compared to other series (39, 40). In the present study, although differences did not reach statistical significance (likely due to the limited number of patients with hypocomplementemia), a higher frequency of low complement levels was observed among patients fulfilling the classification criteria for APS. This finding is consistent with the greater immunological activation in this subgroup since they have a more active serological profile. Regarding the impact of complement on the number of pregnancies and APO, patients with hypocomplementemia showed a higher frequency of PE, which is consistent with some previous studies (11, 41). However, the impact of complement levels was more relevant in the group of patients with APS-NC. In this sense, patients with hypocomplementemia showed a trend towards a lower number of pregnancies, as well as a higher frequency of early abortions and PE. Other series also describe abortions associated with hypocomplementemia (11, 13, 40, 42), and a protective effect of complement inhibition against fetal loss (43). Although the role of hypocomplementemia in fertility has been less extensively characterized than that of ANA, emerging evidence suggests that it may also serve as a predictive factor for reduced pregnancy rates.

Notably, the differences among the APS subgroups studied (primary APS, APS-NS, APS-NC) reveal distinct immunological patterns, which could have diagnostic, prognostic, and therapeutic implications. In particular, the data suggest that APS-NC could even represent an incomplete or progressive form of systemic autoimmunity. Interestingly, serological profiles, such as APS-NC, may provide relevant information in APS. Although some findings in this study did not reach statistical significance, consistent trends were observed that may indicate a potential clinical role for ANA positivity and hypocomplementemia within this subgroup.

Overall, patients who presented any of the two serological alterations more frequently received antimalarial agents. It is worth asking whether this more widespread use of hydroxychloroquine is a response to increased autoimmune activity (cause) or, on the contrary, whether it is used specifically to control such autoimmunity (consequence). In this context, the findings reported by Bertolaccini et al. (36) in murine models are particularly noteworthy, demonstrating that complement inhibition mediates the protective effects of hydroxychloroquine in pregnancy complications associated with APS. Furthermore, although antithrombotic therapy, and in refractory cases the use of antimalarials, remains the cornerstone of obstetric APS management, immunomodulatory treatments may be considered in selected high-risk cases, particularly when pregnancy morbidity appears to reflect broader immune dysregulation. In fact, complement activation, autoantibody-mediated placental injury, cytokine imbalance, and natural killer cell abnormalities have all been implicated in adverse pregnancy outcomes in autoimmune settings (44). In this regard, IVIg has shown promising reproductive results in women with recurrent pregnancy loss associated with APS or other immune disorders, although the indication remains to be better defined (45). This is consistent with recent literature highlighting the need for more individualized and multidisciplinary management strategies in APS (1) (46).

From a clinical perspective, our findings support the potential usefulness of ANA and complement assessment as additional tools for risk stratification in women within the spectrum of obstetric APS. Although these markers are not currently included in APS classification criteria, their association with adverse obstetric outcomes and with a greater use of adjunctive therapies suggests that they may reflect a subgroup of patients with increased immune activation. In daily clinical practice, ANA positivity and hypocomplementemia may help identify women who could benefit from closer multidisciplinary follow-up and consideration of immunomodulatory strategies, in addition to standard antithrombotic treatment.

Our study presents certain limitations. Primarily, those are inherent to their retrospective design. Although ANA and complement levels have been assessed in most patients in recent years, these determinations were not available for all individuals followed in our unit. Additionally, the study was conducted at a single center within a multidisciplinary unit specifically devoted to managing obstetric complications in patients with autoimmune diseases, which may limit the generalizability of the findings. This means that the results cannot be extrapolated to other populations, and probably to the care of pregnant patients outside specialized units. A relevant limitation when investigating the impact of serological alterations on the development of APO is that the analysis is performed on a patient basis and not on pregnancies. Nevertheless, in clinical practice, risk assessment and therapeutic decisions in antiphospholipid-positive women are made based on their cumulative obstetric history, not on isolated pregnancies. Therefore, our patient-level approach not only avoids statistical pitfalls but also mirrors the way these patients are evaluated and managed in real-world settings. Another limitation of this study is that we have not analyzed the main outcomes according to the aPL levels. Finally, other aPL not included in the classification criteria were not analyzed, which could have helped better to categorize the different groups, especially SN-APS and NC-APS (47).

We consider that our study has several advantages over previous ones. Firstly, some studies have been carried out, including patients with aPL associated with other autoimmune diseases, mainly SLE (39, 48, 49), whereas those patients have been excluded from our study. Thus, we could analyze a more homogeneous population of patients belonging to the clinical spectrum of APS. Secondly, the present cohort represents the whole spectrum of patients with a clinical suspicion of APS. It ranges from APS-SN to patients with primary APS, defined according to the classification criteria (1). Moreover, our study includes patients with aPL who present obstetric manifestations not included in these criteria, but that represent a very relevant subgroup in routine clinical practice. Another advantage is that, in addition to the cardiovascular risk factors and the serological profile, we have also assessed other comorbidities that could influence the overall obstetric prognosis (5–7).

In summary, ANA positivity and hypocomplementemia are detectable in a relevant proportion of patients within the clinical spectrum of obstetric APS and were associated with poor obstetric outcomes. The presence of these serological abnormalities was associated with more frequent use of adjunctive antimalarial therapy during pregnancy. Assessment of ANA and complement levels may contribute to improved immunological characterization and risk stratification in patients with suspected obstetric APS.

## Data Availability

The raw data supporting the conclusions of this article will be made available by the authors, without undue reservation.

## Supplementary material

Supplementary Table 1 Study groups according to the clinical and serological manifestations of the Sydney criteria and the presence of obstetric morbidity related to antiphospholipid syndrome (APS);
