# Supplementary material for: The impact of antinuclear antibodies and complement levels in the prognosis of pregnant women within the antiphospholipid syndrome spectrum
Source: Front Immunol. 2026 Jun 5;17:1818605. doi: 10.3389/fimmu.2026.1818605 (PMC13279313; doi:10.3389/fimmu.2026.1818605)
Supplement: Supplementary Table 1 — Study groups according to the clinical and serological manifestations of the Sydney criteria and the presence of obstetric morbidity related to antiphospholipid syndrome (APS); [file Table1.docx]

**Suppl. Table 1.** Study groups according to the clinical and serological manifestations of the Sydney criteria and the presence of obstetric morbidity related to antiphospholipid syndrome (APS).

| SEROLOGY | CLINICAL MANIFESTATIONS | | |
| --- | --- | --- | --- |
|  | ***SIDNEY CRITERIA*** | ***RELATED OBSTETRIC MORBIDITY*** | ***NO MANIFESTATIONS*** |
| *SIDNEY CRITERIA* | CRITERIA APS  (N=85) | SUBGROUP C  (N=62) | - |
| *INCONCLUSIVE* | SUBGROUP B  (N=50) | SUBGROUP A  (N=27) | - |
| *NEGATIVE* | SERONEGATIVE APS  (N=61) | - | - |

Non-Criteria APS (N=139) includes subgroups A, B and C.
